# The Doctor’s Wife

**Published:** 1905-07

**Authors:** 


### The Doctor's Wife.

BY V. A. H. D.

Do you know, it seems to me
  A little bit strange, sometimes,
That the wives of medical men
  Never have their virtues defined;

Now we hear through song and rhyme
  Of "the good old country doctor,"
But is there ever a time
  When we hear of his wife or daughter?

Now tell me, for really I mean it,
  Are her praises ever sung?
By writer, or songster, or poet—
  To the tune of aught but tongue?

I dwell on this fact with regret,
  For what would he be without her?
For to her he may fume and fret
  How others neglect his orders!

When he comes in cross and cold
  And hungry, of course, she expects;
Then she must not make so bold
  As to ask him "why" he frets!

She must have the "meals on time,"
  E'en should he be hours late;
And all "must be hot" and fine,
  No matter the poor cook's fate!

She must bright and smiling be,
  For he's "tired of frowns and complaints,"
And he wants, just between you and me,
  Nothing short of the virtue of saints!

When he sits down to meals, who's to keep
  All the people from knowing he's there?
When his buggy stands out in the street,
  And the people seek him in despair?

How can she "tell central" to hush,
  That she's going to "drop the receiver!"
When it might be a child, sorely hurt,
  Or a mother needing him to relieve her!

And then, if he's on some committee,
  Or member of some association,
Who is it that on him takes pity,
  And sits up to "re-hash" his oration?

For don't think for a moment, my dears,
  That his wife doesn't "back him up" in it!
For after he promised, his fears
  Set in at one hundred per minute!

And he thinks that he "never can write it,"
  That paper he "promised in fun!"
Then she has to coax and encourage
  And smooth it, in more ways than one!

So, here's to the doctor's wife!
  Whether he uses a "gig" or an "auto,"
For she leads "the strenuous life."
  All for the love of him, I know!

To the wives of the bravest of men!
  Who stand by their husband's side
To love and obey to the end,
  Though often their spirits are tried!

---

THE EAST TEXAS MEDICO-CHIRURGICAL ASSOCIATION did not hold its regular May meeting on account of illness of certain active members, but will meet in November. Notice of date and place will be announced later. Dr. W. B. Collins, Lovelady, is President, and Dr. W. B. Ramsay, of Alto, is Secretary,

---

THE PASSING OF THE WHISKERS.—The State Board of Health of Iowa have issued orders "commanding all doctors to be shaved," says *Medical Notes and Queries.*

